# Effects of Heme Oxygenase-1 Upregulation on Blood Pressure and Cardiac Function in an Animal Model of Hypertensive Myocardial Infarction

**DOI:** 10.3390/ijms14022684

**Published:** 2013-01-28

**Authors:** Tian-meng Chen, Jian Li, Lin Liu, Li Fan, Xiao-ying Li, Yu-tang Wang, Nader G. Abraham, Jian Cao

**Affiliations:** 1 First Department of Geriatric Cardiology, Chinese PLA General Hospital, Beijing 100853, China; E-Mails: mengminami@yahoo.com.cn (T.C.); li_yinh@yahoo.com.cn (J.L.); bjll715@126.com (L.L.); fl6698@163.com (L.F.); lixy301@yahoo.com.cn (X.L.); wyt301@sina.com (Y.W.); 2 School of Medicine, Nankai University, Tianjin 300071, China; 3 Marshall University Joan C. Edward School of medicine, 1600 Medical Center Drive, Huntington, WV 25701, USA; E-Mail: abrahamn@Marshall.edu

**Keywords:** hypertension, myocardial infarction, heme oxygenase, bilirubin

## Abstract

In this study, we evaluate the effect of HO-1 upregulation on blood pressure and cardiac function in the new model of infarct spontaneous hypertensive rats (ISHR). Male spontaneous hypertensive rats (SHR) at 13 weeks (*n* = 40) and age-matched male Wistar (WT) rats (*n* = 20) were divided into six groups: WT (sham + normal saline (NS)), WT (sham + Co(III) Protoporphyrin IX Chloride (CoPP)), SHR (myocardial infarction (MI) + NS), SHR (MI + CoPP), SHR (MI + CoPP + Tin Mesoporphyrin IX Dichloride (SnMP)), SHR (sham + NS); CoPP 4.5 mg/kg, SnMP 15 mg/kg, for six weeks, one/week, i.p., *n* = 10/group. At the sixth week, echocardiography (UCG) and hemodynamics were performed. Then, blood samples and heart tissue were collected. Copp treatment in the SHR (MI + CoPP) group lowered blood pressure, decreased infarcted area, restored cardiac function (left ventricular ejection fraction (LVEF), left ventricular fraction shortening (LVFS), +d*p*/d*t*_max_, (−d*p*/d*t*_max_)/left ventricular systolic pressure (LVSP)), inhibited cardiac hypertrophy and ventricular enlargement (downregulating left ventricular end-systolic diameter (LVEDD), left ventricular end-systolic diameter (LVESD) and heart weight/body weight (HW/BW)), lowered serum CRP, IL-6 and Glu levels and increased serum TB, NO and PGI2 levels. Western blot and immunohistochemistry showed that HO-1 expression was elevated in the SHR (MI *+* CoPP) group, while co-administration with SnMP suppressed the benefit functions mentioned above. In conclusion, HO-1 upregulation can lower blood pressure and improve post-infarct cardiac function in the ISHR model. These functions may be involved in the inhibition of inflammation and the ventricular remodeling process and in the amelioration of glucose metabolism and endothelial dysfunction.

## 1. Introduction

Primary hypertension is a major risk factor of cardiovascular diseases, including cardiomyopathy, coronary artery diseases or even heart failure. Moreover, hypertension also tends to aggravate clinical courses, leading to higher incidence of cardiogenic shock, pulmonary edema, sinus tachycardia on admission, ventricular tachycardia or fibrillation, complete A-V block and in-hospital deaths. In other words, patients with both ST-elevated myocardial infarction (STEMI) and hypertension tend to have more cardiovascular risk factors and suffer a more complicated in-hospital course of MI than normotensive patients [1].

Heme oxygenase (HO), a rate-limiting enzyme in heme metabolism, degrades heme into biliverdin/bilirubin, with the production of carbon monoxide (CO) and free iron (Fe). The products of heme metabolism, including bilirubin, CO and Fe, possess various beneficial physiological functions, such as anti-oxidant stress, anti-apoptosis, anti-inflammation, vasodilation, regulating cell proliferation, enhancing insulin sensitivity, inducing adiponectin and regulating angiogenesis [2]. Heme oxygenases mainly include two isoenzymes, HO-1 and HO-2. HO-1 is an inducible isoenzyme, whose expression and activity can be upregulated or downregulated by inducers or inhibitors. HO-1 is mainly distributed in spleen and in reticuloendothelial cells of liver or bone marrow, while seldom in the organs with little relevance to senescent erythrocytes degradation. In these organs, HO-1 can be upregulated in the form of elevated protein expression or enhanced activity [3]. HO-2 is a constitutive enzyme, widely spread in almost all the organs of human beings, but cannot be induced [2]. In consideration of the inducible character and potentially wide distribution, HO-1 has been regarded as the central part in the HO system.

In the treatment of metabolic syndrome, upregulating HO-1 expression and activity is a new focus for both basic and clinical studies. It also seems to be a promising therapeutic approach [4]. It has been reported that SHR exhibited obvious endothelial dysfunction (reduction of NO- and EDHF-mediated vasodilatation [5]), impaired NO availability and excessive oxidative stress. HO-1 upregulation by the inducers improved NO levels, attenuated oxidative stress injury, suppressed smooth muscle cell (VMC) proliferation, along with endothelial function promotion, vessel dilation and blood pressure declination [6,7]. Cardiac hypertrophy was one of the major complications associated with hypertension, partially resulting from undue inflammation and oxidative stress. HO-1 upregulation also relieved cardiac hypertrophy, in view of the reduced left-to-right ventricular ratio, left ventricular wall-thickness, ventricle-to-weight ratio and extracellular matrix/remodeling proteins, along with the attenuated cardiac histopathological lesions. The underlying mechanisms should be attributed to the inhibition of inflammation and oxidative reaction [8–10]. From the perspective of myocardial infarction, upregulation of HO-1 activity and expression, no matter which kind of method was adopted, were all able to reduce infarct size, suppress ventricular remodeling and ameliorate post-infarct cardiac function, through the mechanisms of anti-apoptosis and anti-inflammation [11–15].

Based on these available experimental evidences, we postulated that HO-1 upregulation in a new model, infarct spontaneous hypertensive rat (ISHR), could also bring in a cardioprotective adaption in the way of suppressing elevated blood pressure, ameliorating pathological ventricular remodeling and improving hypertensive post-infarct cardiac function.

## 2. Results and Discussion

### 2.1. Results

#### 2.1.1. Baseline Characters between WT and SHR

SHRs exhibited significantly higher mean arterial (MAP), systolic (SBP) and diastolic blood pressure (DBP) compared to WTs. There was no statistical significance in heart rate (HR) between SHRs and WTs (Table 1).

SHRs exhibited significantly smaller left ventricular end-diastolic and end-systolic diameter (LVEDD and LVESD) compared to WTs. Echocardiographic images showed obvious cardiac hypertrophy in SHRs. However, there was no statistical significance in left ventricular ejection fraction (LVEF) and left ventricular fraction shortening (LVFS) between SHRs and WTs (Table 2).

#### 2.1.2. CoPP Upregulated HO-1 Expression in ISHR Model

**Figure 1:**
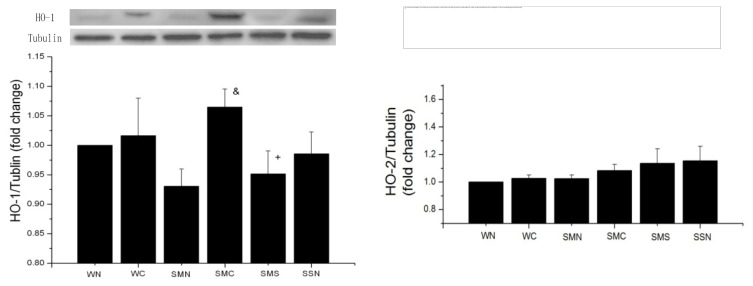
HO-1 and HO-2 expression in heart tissue of wistar rats (WTs) and spontaneous hypertensive rat (SHRs) at sixth week after operation (Western blot). ^&^*p* < 0.05 *vs.* SMN; ^+^*p* < 0.05 *vs.* SMC. WN: WT (sham + NS); WC: WT (sham + CoPP); SMN: SHR (MI + NS); SMC: SHR (MI + CoPP); SMS: SHR (MI + CoPP + SnMP); SSN: SHR (sham + NS). *n* = 5 for each group.

Basic HO-1 protein expression levels were listed from the top to the end as below: WT (sham + NS) > SHR (sham + NS) > SHR (MI + NS), while there was no statistical significance among these three groups (*p* > 0.05). CoPP treatment for six weeks resulted in HO-1 upregulation in the SHR (MI + CoPP) group (*p* < 0.05), while concurrent SnMP treatment blocked HO-1 protein overexpression. HO-2 protein levels were unaffected, despite different states in blood pressure, myocardial infarction and medication treatment among the groups (Figure 1).

**Figure 2:**
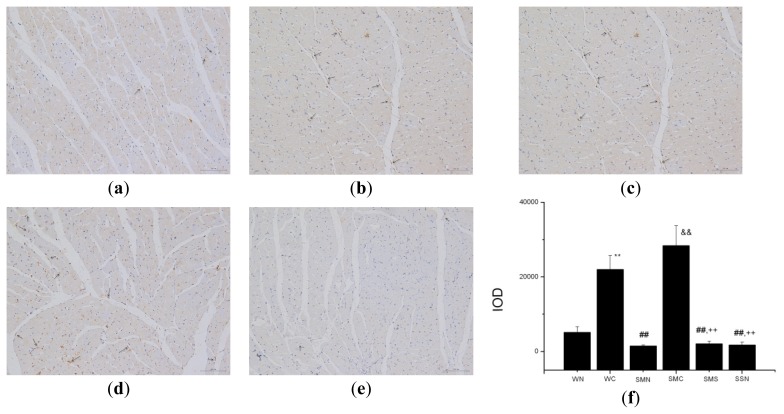
HO-1 expression in heart tissue of WTs and SHRs at sixth week after operation (immunochemistry). (**a**) WT (sham + NS); (**b**) WT (sham + CoPP); (**c**) SHR (MI + NS); (**d**) SHR (MI + CoPP); (**e**) SHR (MI + CoPP + SnMP); (**f**) IOD (integral optical density) values among six groups. **p < 0.01 *vs.* WN; ^##^*p* < 0.01 *vs.* WC; ^&&^*p* < 0.01 *vs.* SMN; ^++^*p* < 0.01 *vs.* SMC. WN: WT (sham + NS); WC: WT (sham + CoPP); SMN: SHR (MI + NS); SMC: SHR (MI + CoPP); SMS: SHR (MI + CoPP + SnMP); SSN: SHR (sham + NS). *n* = 7 for each group.

The result of immunohistochemistry was similar to that of Western blot, showing that CoPP treatment for six weeks could upregulated HO-1 expression in both WT (sham + CoPP) and SHR (MI + CoPP) groups (*p* < 0.01). Besides, the upregulation of HO-1 expression was more obvious in the latter one (*p* > 0.05). Concurrent SnMP treatment could inhibit HO-1 protein overexpression (Figure 2).

#### 2.1.3. CoPP Treatment Enhanced Serum Total Bilirubin Levels

**Figure 3:**
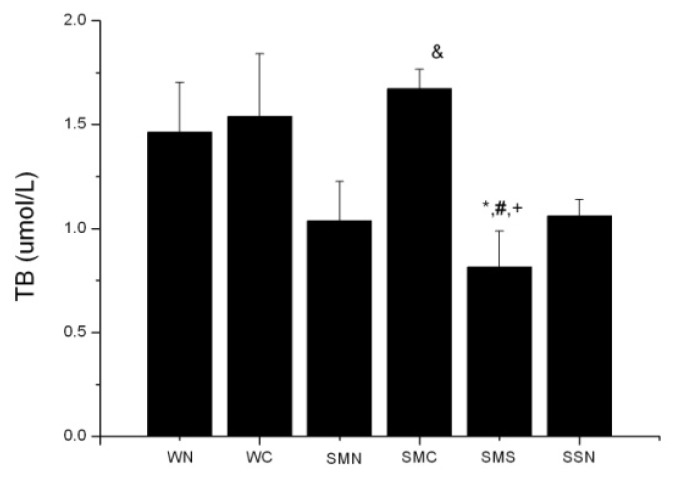
Serum bilirubin levels of WTs and SHRs at sixth week after operation. *p < 0.05 *vs.* WN; ^#^*p* < 0.05 *vs.* WC; ^&^*p* < 0.05 *vs.* SMN; ^+^*p* < 0.0 *vs.* SMC. WN: WT (sham + NS); WC: WT (sham + CoPP); SMN: SHR (MI + NS); SMC: SHR (MI + CoPP); SMS: SHR (MI + CoPP + SnMP); SSN: SHR (sham + NS). *n* = 8 for each group.

Bilirubin is one of the three main metabolic products in the HO system. Serum bilirubin levels could, to some extent, reflect HO activity. There was no statistical difference between WT (sham + NS) and SHR (MI + NS) groups, in spite of a slightly lower level in the latter one (*p* > 0.05). CoPP treatment for six weeks elevated serum total bilirubin levels in the SHR (MI + CoPP) group (*p* < 0.05), while concurrently treating with SnMP could block this effect (Figure 3).

#### 2.1.4. CoPP Treatment Reduced Blood Pressure in SHR

**Figure 4:**
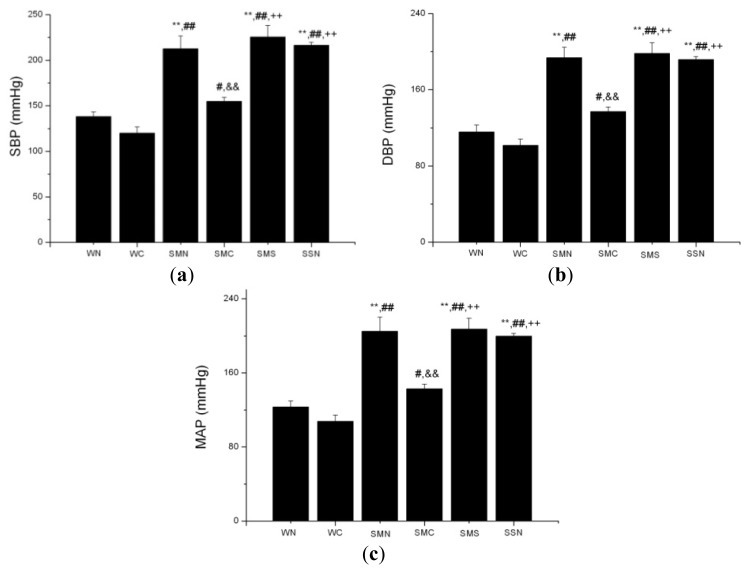
Blood pressure of WTs and SHRs at 6th week after operation. (**a**) SBP; (**b**) DBP; (**c**) MAP. *p < 0.05; **p < 0.01 *vs.* WN; ^#^*p* < 0.05; ^##^*p* < 0.01 *vs.* WC; ^&^*p* < 0.05; ^&&^*p* < 0.01 *vs.* SMN; ^+^*p* < 0.05; ^++^*p* < 0.01 *vs.* SMC; ^$^*p* < 0.05; ^$$^*p* < 0.01 *vs.* SMS. WN: WT (sham + NS); WC: WT (sham + CoPP); SMN: SHR (MI + NS); SMC: SHR (MI + CoPP); SMS: SHR (MI + CoPP + SnMP); SSN: SHR (sham + NS). *n* = 8 for each group.

CoPP treatment significantly reduced elevated blood pressure, including SBP, DBP and MAP in SHRs (*p* < 0.001), while concurrently treating with SnMP could block this effect (Figure 4).

#### 2.1.5. CoPP Treatment Ameliorated Endothelial Dysfunction

The SHR (MI + NS) group had lower serum NO levels (*p* < 0.05). CoPP treatment resulted in the increased serum NO levels in the SHR (MI + CoPP) group (*p* < 0.01) compared with the SHR (MI + NS) group.

Serum PGI_2_ levels showed no statistical difference between the SHR (MI + NS) and WT (sham + NS) group. CoPP treatment increased serum PGI_2_ levels in both the WT (sham + CoPP) (*p* < 0.05) and SHR (MI + CoPP) (*p* < 0.01) group. In addition, the increased range was more obvious in the SHR (MI + CoPP) group compared with the WT (sham + CoPP) group.

**Figure 5:**
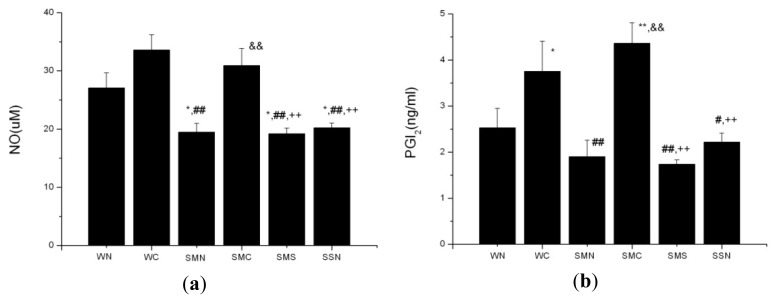
Serum NO and PGI_2_ levels of WTs and SHRs at sixth week after operation. (**a**) NO; (**b**) PGI_2_. *p < 0.05; **p < 0.01 *vs.* WN; ^#^*p* < 0.05; ^##^*p* < 0.01 *vs.* WC; ^&&^*p* < 0.01 *vs.* SMN; ^++^*p* < 0.01 *vs.* SMC. WN: WT (sham + NS); WC: WT (sham + CoPP); SMN: SHR (MI + NS); SMC: SHR (MI + CoP*P*); SMS: SHR (MI + CoPP + SnMP); SSN: SHR (sham + NS). *n* = 8 for each group.

Concurrently, SnMP treatment in the CoPP-treated ISHR model markedly suppressed the elevated serum NO and PGI_2_ levels (*p* < 0.01) (Figure 5).

#### 2.1.6. CoPP Treatment Diminished Infarct and Peri-Infarct Area in ISHR Model

Compared to the WT (sham + NS) group, the SHR (MI + NS) group exhibited obvious myocardial infarction. Masson’s trichrome stain showed that the majority of myocytes in the infarct area disappeared and were replaced by fibrosis. HE stain showed that in a peri-infarct area existed myocyte hypertrophy, hyperemia and chronic inflammation. CoPP treatment reduced infarct area enlargement and its fibrosis progress. CoPP also inhibited myocyte hypertrophy, hyperemia and chronic inflammation in the peri-infarct area. Concurrently treating with SnMP could block all these functions mentioned above (Table 3).

#### 2.1.7. CoPP Treatment Inhibited Ventricular Remodeling of Post-Infarction in SHR

**Figure 6:**
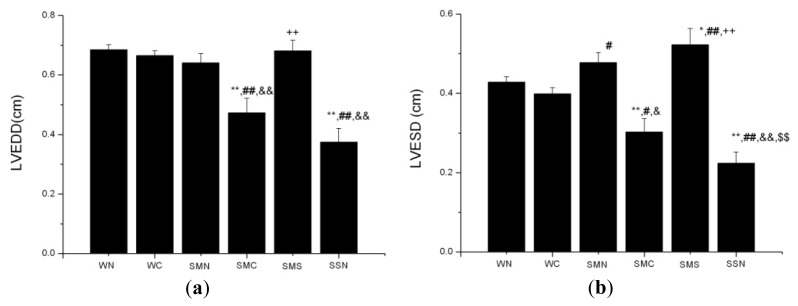

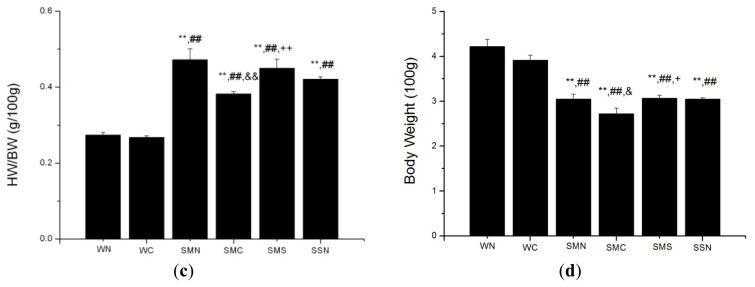
LVEDD, LVESD and HW/BW values of WTs and SHRs at sixth week after operation. (**a**) LVEDD; (**b**) LVESD; (**c**) HW/BW; (**d**) BW. *p < 0.05; **p < 0.01 *vs.* WN; ^#^*p* < 0.05; ^##^*p* < 0.01 *vs.* WC; ^&^*p* < 0.05; ^&&^*p* < 0.01 *vs.* SMN; ^+^*p* < 0.05; ^++^*p* < 0.01 *vs.* SMC; ^$^*p* < 0.05; ^$$^*p* < 0.01 *vs.* SMS. WN: WT (sham + NS); WC: WT (sham + CoPP); SMN: SHR (MI + NS); SMC: SHR (MI + CoPP); SMS: SHR (MI + CoPP + SnMP); SSN: SHR (sham + NS). *n* = 8 for each group.

**Figure 7:**
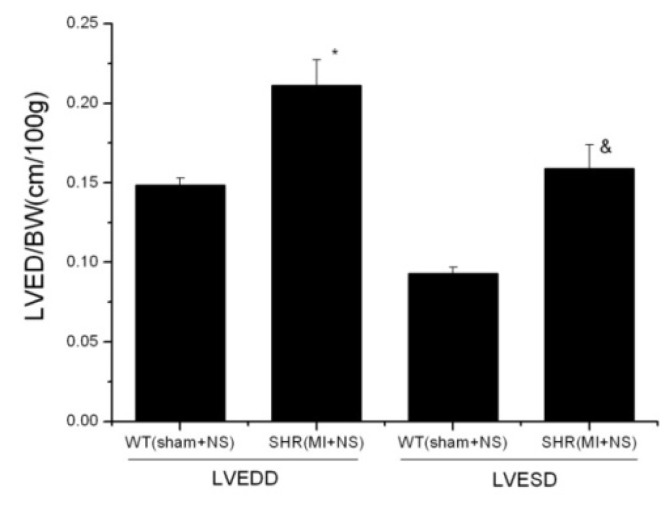
LVEDD, LVESD adjusted for body weight. * WT (sham + NS) *vs.* SHR (MI + NS) *p* = 0.001; ^&^ WT (sham + NS) *vs.* SHR (MI + NS) *p* < 0.001. *n* = 8 for each group.

The SHR (MI + NS) group exhibited significantly increased LVEDD and LVESD (*p* < 0.001) compared with SHR (sham + NS), while no statistical significance existed in LVEDD and LVESD between the WT (sham + NS) group and the SHR (MI + NS) group. CoPP treatment inhibited the increase of LVEDD and LVESD in the SHR (MI+ CoPP) group (*p* < 0.001). There was no statistical difference in LVEDD and LVESD between the WT (sham + NS) and WT (sham+ CoPP) group. After the adjustment for body weight (BW) (Figure 6), the values of LVEDD/BW and LVESD/BW in the SHR (MI + NS) group were both significantly higher than those in the WT (sham + NS) group (*p* < 0.001) (Figure 7).

Compared with WT, SHR had a much higher ratio of heart weight to body weight (HW/BW) (*p* < 0.001). CoPP treatment significantly reduced the elevated HW/BW ratio in the SHR (MI + CoPP) group (*p* = 0.001), which was still obviously higher than that in WT groups (*p* < 0.001) (Figure 6).

Concurrently, SnMP treatment markedly suppressed the CoPP functions mentioned above, including the inhibition of the enlargement of LVEDD and LVESD and the reduction of the HW/BW ratio (*p* < 0.01) (Figure 6).

#### 2.1.8. CoPP Treatment Improved Cardiac Function of Post-Infarction in SHR

**Figure 8:**
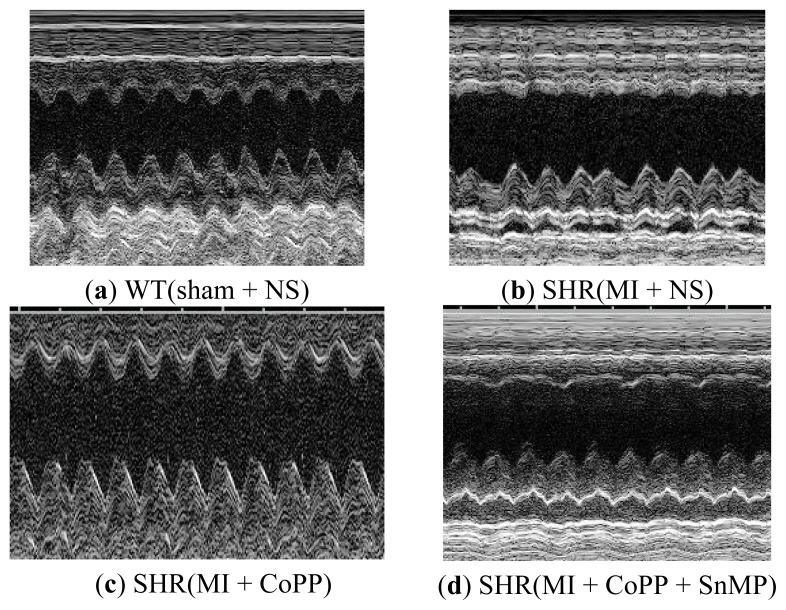
Echocardiographic images of WTs and SHRs at sixth week after operation. (**a**) WT (sham + NS) group; (**b**) SHR (MI + NS) group; (**c**) SHR (MI + CoPP); (**d**) SHR (MI + CoPP + SnMP) group.

Six weeks after left anterior descending coronary artery ligation, LVEF and LVFS in the SHR (MI + NS) group were significantly lower than those in WT and SHR under sham operation (*p* < 0.001). CoPP treatment improved the exacerbated LVEF and LVFS in the SHR (MI + CoPP) group (*p* < 0.001). Concurrent administration of SnMP with CoPP in the SHR (MI + CoPP + SnMP) group prevented the amelioration of LVEF and LVFS (Figure 8 and Figuer 9).

Compared to the WT (sham + NS) group, the SHR (MI + NS) group exhibited higher LVSP and LVEDP, lower +d*p*/d*t*_max_ (*p* < 0.01), while −d*p*/d*t*_max_ levels showed no statistical significance between these two groups (*p* > 0.05). After the adjustment for LVSP, (−d*p*/d*t*_max_)/LVSP in the SHR (MI + NS) group was significantly lower than that in the WT (sham + NS) group (*p* < 0.01).

**Figure 10:**
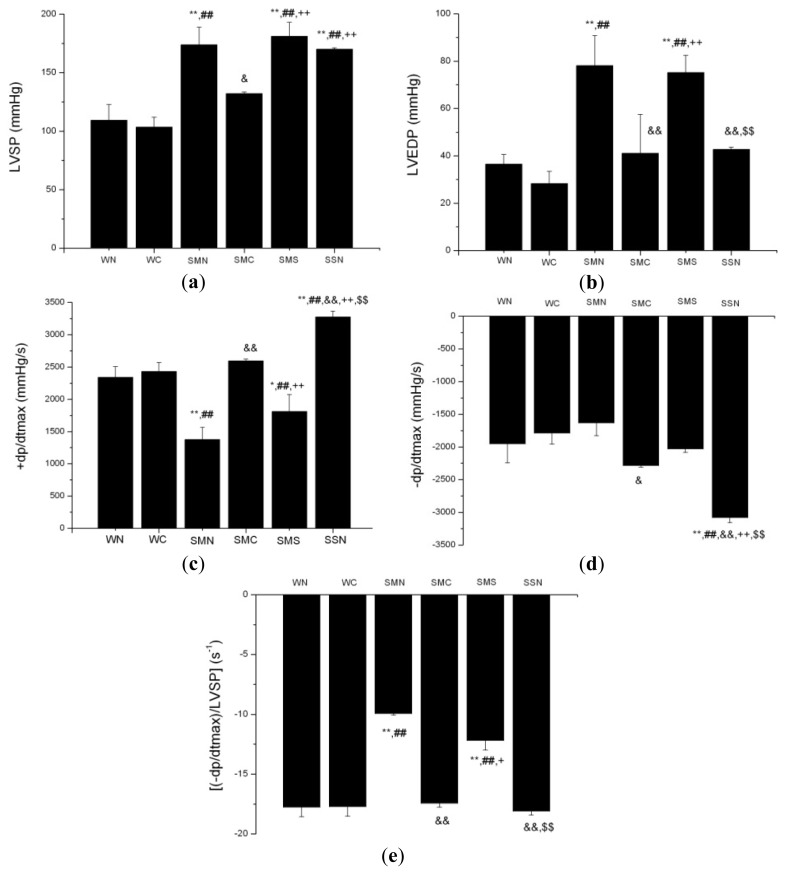
Hemodynamic parameters of WTs and SHRs at sixth week after operation. (**a**) LVSP; (**b**) LVEDP; (**c**) +d*p*/d*t*_max_; (**d**) −d*p*/d*t*_max_; (**e**) (−dp/dtmax)/LVSP. *p < 0.05; **p < 0.01 *vs.* WN; ^#^*p* < 0.05; ^##^*p* < 0.01 *vs.* WC; ^&^*p* < 0.05; ^&&^*p* < 0.01 *vs.* SMN; ^+^*p* < 0.05; ^++^*p* < 0.01 *vs.* SMC; ^$^*p* < 0.05; ^$$^*p* < 0.01 *vs.* SMS. WN: WT (sham + NS); WC: WT (sham + CoPP); SMN: SHR (MI + NS); SMC: SHR (MI + CoPP); SMS: SHR (MI + CoPP + SnMP); SSN: SHR (sham + NS). *n* = 8 for each group.

CoPP treatment decreased LVSP and LVEDP, increased +d*p*/d*t*_max_, −d*p*/d*t*_max_ and (−dp/dtmax)/LVSP (*p* < 0.05 or *p* < 0.01) in the SHR (MI + CoPP) group compared with the SHR (MI + NS) group. However, concurrent administration of SnMP prevented these functions of CoPP treatment. CoPP treatment made no differences in LVSP, LVEDP, +d*p*/d*t*_max_, −d*p*/d*t*_max_ and (−d*p*/d*t*_max_)/LVSP between the CoPP treated and untreated group in WTs (Figure 10).

#### 2.1.9. CoPP Treatment Suppressed Inflammatory Cytokines Levels

**Figure 11:**
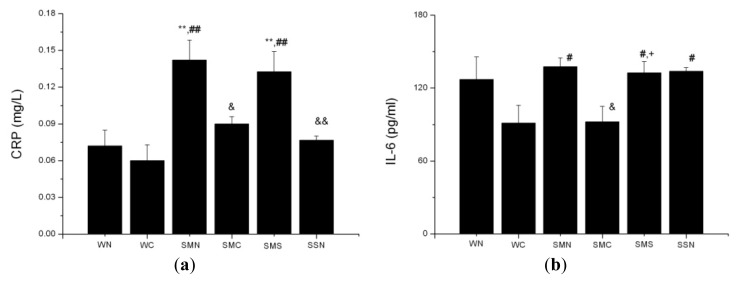
Serum CRP and IL-6 levels of WTs and SHRs at sixth week after operation. (**a**) CRP; (**b**) IL-6. **p < 0.01 *vs.* WN; ^#^*p* < 0.05; ^##^*p* < 0.01 *vs.* WC; ^&^*p* < 0.05 *vs.* SMN; ^+^*p* < 0.05 *vs.* SMC. WN: WT (sham + NS); WC: WT (sham + CoPP); SMN: SHR(MI + NS); SMC: SHR (MI + CoPP); SMS: SHR (MI + CoPP + SnMP); SSN: SHR (sham + NS). *n* = 8 for each group.

The SHR (MI + NS) group exhibited higher C-reactive protein (CRP) levels compared with the WT (sham + NS) group (*p* < 0.01). CoPP treatment decreased the elevated CRP levels in the ISHR model (*p* < 0.05), while concurrent SnMP administration blocked this decrease. Similar to the pattern observed with CRP, basal levels of serum IL-6 were a little higher in the SHR (MI + NS) group than in the WT(sham + NS) group, but with no statistical significance (*p* > 0.05). CoPP treatment also suppressed IL-6 levels in the ISHR model (*p* < 0.05) (Figure 11).

### 2.2. Discussion

In this study, we demonstrate for the first time that HO-1 upregulation through CoPP administration in a new model, infarct spontaneous hypertensive rats (ISHR), possesses a cardiovascular protective function that attenuates blood pressure, alleviates pathological left ventricular remodeling and ameliorates post-infarct cardiac function. Several lines of evidence support these conclusions. First, the results of Western blot and immunochemistry showed that HO-1 expression was upregulated and that the levels of bilirubin, one product of the HO system, were also elevated after CoPP administration. Second, meanwhile, CoPP treatment also suppressed elevated blood pressure, LV dilatation, hypertrophy, inflammatory reaction and endothelial and cardiac dysfunction. Third, concurrently SnMP administration, an HO activity inhibitor, inhibited HO-1 overexpression, reduced serum bilirubin levels and blocked all the CoPP effects mentioned above, which further suggested that all these CoPP cardiovascular protective effects should be ultimately attributed to HO-1 upregulation. However, some studies also showed that SnMP could only suppress HO activity rather than HO-1 expression [16]. We were also puzzled by these results. These different phenomena might be due to the following issues. The first reason was that the animals concurrently treated with SnMP were very weak or even dying when the experiment duration ended. Also, the terminal stage with multiple organ failure might lead to the impaired synthesis, degradation or even exhaustion of the HO-1 protein. Other reasons for lower expression of HO-1 by SnMP treatment might be associated with fatal inflammation, endothelial dysfunction, excessive oxidation, and so on. It has been reported that HO-1 protein in the kidneys of WKY rats with diabetes was significantly less than that of the WKY control groups [17]. This downregulation of HO-1 protein might be due to the excessive oxidation and inflammation resulting from diabetes. In our study, myocardial infarction and hypertension were both two strong pathophysiological stimulations. Also, the administration of SnMP blocked HO activity, possibly including not only HO-1 activity from CoPP upregulation, but also that of the baseline, which would further aggravate the pathophysiological situations of the SHR(MI + CoPP + SnMP) group. In this way, the downregulated effect on HO-1 protein expression of severe pathophysiological situations might overwhelm the upregulation of SnMP on HO-1 expression. However, we need further study to prove this in the future. In spite of this inconsistent evidence, the inhibition of HO activity by SnMP treatment has been demonstrated. These results indicated a cardioprotective role (anti-hypertrophy, anti-LV remodeling, anti-inflammation), a vasculoprotective role (anti-hypertension, anti-endothelial dysfunction) and a potential therapeutic effect of enhancing HO-1 function in patients with both hypertension and myocardial infarction. These findings indicated that augmentation of HO-1 may be a novel and beneficial therapeutic approach in hypertensive myocardial infarct patients.

#### 2.2.1. HO-1 Attenuation of Blood Pressure and Amelioration of Endothelial Dysfunction

The anti-hypertensive effect of HO-1 has already been acknowledged. In a basic experiment, the SHR model exhibited an obvious decrease in HO-1 expression levels compared with WKY [18], indicative of HO-1 system dysfunction in hypertension. Upregulating the activity and expression of HO-1 could inhibit the process of hypertension [17,19], which coincides with our results. The associated mechanisms involve restoration of endothelial function [17], inhibition of VSMC proliferation [20], suppression of kidney oxidative and inflammatory injury [21], amelioration of vascular regulative factors [22,23] and direct relaxation of blood vessels by CO [23].

It has been generally accepted that hypertension is closely associated with endothelial dysfunction (elevated vascular resistance and reduced sensitivity to stimuli of vasodilation) in the peripheral coronary and renal circulations. NO is a well-known vital autocrine and paracrine signaling molecule in the regulation of several cell functions, including modulation of vasomotor tone [24], inhibition of SMC proliferation and migration, leukocyte adhesion and platelet aggregation [25]. In an *in vitro* study, adenosine-stimulated NO-release was obviously impaired in hypertensive vessels compared to normotensive vessels [26]. Our study also showed the inferior NO basal levels in the ISHR model *in vivo*. Both results demonstrated that the inhibited NO production was closely associated with the development of hypertension. Up to now, the role of NO in SHR still remains to be the subject of debate. Impaired, unchanged or enhanced NO function all have been reported in SHR. Despite these inconsistencies, the existence of impaired NO bioavailability in SHR has been shown [22]. In this study, the amelioration of blood pressure in the ISHR model by HO-1 upregulation, to some extent, should be attributed to the elevated NO levels.

Prostanoids have also been regarded as another important factor in the regulation of blood pressure. In addition to being a potent vasodilator, PGI_2_ is also a mediator of renin secretion, which in turn will lead to the development of renovascular hypertension [27]. In this way, the exact effect of PGI_2_ on hypertension, alleviation or aggravation, is still in doubt.

#### 2.2.2. HO-1 Modulation of Post-Infarction Pathological LV Remodeling

In the ISHR model, hypertension increased the afterload. Meanwhile, myocardial infarction reduced cardiac output. Both initiated the cardiac compensatory mechanism, including the Frank-Starling mechanism, ventricular remodeling and neurohormonal action. Ventricular remodeling involves changes in left ventricular size, shape and mass to maintain adequate forward flow, in response to myocyte loss after a myocardial infarction and to hemodynamic overload induced by hypertension. The initial response to increased cardiac stress or load is usually concentric hypertrophy, the myocytes predominantly increasing in width and the heart tending to thicken. According to Laplace’s Law, increased wall thickness could offset the afterload-induced increase in systolic wall stress [28]. However, it will also aggravate increased filling pressure. When the increase occurs mainly in cell length—eccentric hypertrophy—the predominant form of remodeling is ventricular dilation. It will help maintain cardiac output, but at the expense of increased ventricular wall stress. If the extent of hypertrophy is inadequate to normalize wall stress, then a vicious cycle is established. Overstretching of the myocytes can lead to an increase in myocyte death, ventricular dilation, development of a spheric left ventricular cavity and further elevation in wall stress.

In this study, the levels of LVEDD and LVESD showed no difference between the CoPP treated ISHR group and the WT control group, seemingly suggesting that no LV dilation existed after the LAD ligation operation six weeks later. In fact, an important factor, the different growing curves of WTs and SHRs in length and weight, have been neglected. The length and weight in WTs tend to increase more obviously along with the accumulation of age compared with SHRs. Considering these differences, a previous study adopted an adjusted value, the ratio of LVDD/BW [29], to diminish the disturbances and to reach a more scientific conclusion. In this study, after the adjustment for body weight, the SHR (MI + NS) group exhibited a significant increase in LVEDD/BW and LVESD/BW compared to the WT (sham + NS) group. Besides, a significantly smaller LVEDD and LVESD of SHR in baseline echocardiograph and the similar LVDD between the SHR (MI + NS) and WT (sham + NS) group after operation six weeks all further support the existence of LV dilation in the ISHR model. HO-1 upregulation obviously decreased LVDD in the SHR (MI + CoPP) group, but not so in the WT (sham + CoPP) group, indicating HO-1 upregulation could inhibit LV pathological dilation in the ISHR model.

Heart weight/body weight (HW/BW), a heart mass index, is usually used in evaluating the degree of hypertrophy. In this study, the ISHR model exhibited a significantly higher HW/BW ratio, which should be attributed to two main factors, the increased afterload induced by hypertension and the impaired cardiac function caused by myocardial infarction. HO-1 upregulation significantly reduced the HW/BW ratio in the ISHR model, but this ratio in WT groups was unaffected. HE and Masson’s stain also showed the attenuated hypertrophy and chronic inflammation in the peri-infarct area in the CoPP treated ISHR model. This evidence all indicated that HO-1 upregulation could suppress the hypertrophy process in the ISHR model.

Relative studies showed an important status of inflammation in the LV remodeling process [30]. CRP, a cytokine downstream of TNF-α and IL-6 in the inflammatory cascade and a marker of chronic inflammation [31], also plays an inevitable role in ventricular remodeling. In a clinical study, CRP was positively associated with left ventricular mass index (LVW/BW) in hypertensive patients [32]. On one hand, in a basic experiment, CRP could aggravate apoptosis in hypoxia-stimulated myocytes through the mitochondrion-dependent pathway [33]. CRP transgenic mice also showed more LV dilation and worse LV function with more obvious cardiomyocyte hypertrophy and fibrosis in the non-infarct regions after MI than controls [34]. On the other hand, administration of a CRP inhibitor abrogated the increased infarct size and decreased cardiac dysfunction produced by injection of human CRP [35]. In this way, a reciprocal causal relationship exists between CRP and ventricular remodeling, indicating CRP might be a critical factor in the development and progress of ventricular remodeling and also be a central target in the treatment to disturb this vicious cycle.

PGI_2_, produced by the vascular wall and predominantly by the endothelia, acts as a physiological antagonist of TXA_2_. Moreover, PGI_2_ is a powerful cytoprotective agent, exerting its action through activation of adenylate cyclase, followed by an intracellular accumulation of cyclic-AMP in various types of cells [36]. Several reports suggested that PGI_2_ and its receptor (IP) both take the indispensable role in the cardiac hypertrophy process. In the constricted transverse aorta mouse model, the HW/BW ratio in IP−/− mice was significantly greater than those in wild-type mice two or four weeks after operation, with cardiomyocyte cross-sectional area and cardiac fibrosis also obviously augmented [27,37]. In this way, PGI_2_-IP is another important factor associated with prevention of ventricular remodeling. Upregulation of PGI_2_-IP levels is also an available choice in slowing its process.

In this study, HO-1 upregulation ameliorated the levels of both two ventricular remodeling associated factors, decreasing CRP levels and increasing PGI_2_ levels. Besides, it also reduced elevated blood pressure, infarct regions and improved cardiac function. In other words, HO-1 upregulation not only promotes anti-remodeling factors, but also removes pro-remodeling factors, so that it can prevent the development of ventricular remodeling more thoroughly.

#### 2.2.3. HO-1 Amelioration of Hypertensive Post-Infarction Cardiac Function

In this study, echocardiography showed that HO-1 upregulation significantly improved LVEF and LVFS, in other words, the cardiac systolic function in ISHR model. In the hemodynamic perspective, HO-1 upregulation suppressed LVSP and LVEDP, while ameliorating ±d*p*/d*t*_max_ and (−d*p*/d*t*_max_)/LVSP. LVSP, to some extent, is positively correlated to systolic function. The higher the LVSP, the better the cardiac contraction. LVEDP indirectly reflects cardiac preload and is inversely correlated to systolic function, which means the lower, the better. From this point of view, it seems that HO-1 upregulation failed to improve or even exacerbate the systolic function. However, one point needs to be considered, that is, the significant differences in baseline blood pressure between SHR and WT and the dispensable disturbances of different blood pressure to the analysis of LVSP and LVEDP. With the influence of the unbalanced baseline blood pressure, the obviously elevated LVSP and LVEDP in the ISHR model should be mainly attributed to the elevated blood pressure in SHR and failed to correctly reflect the objective cardiac function.

±d*p*/d*t*_max_ are much more accurate indices in discussing cardiac systolic and diastolic function. −d*p*/d*t*_max_, a cardiac diastolic index, stands for the maximal declining velocity of pressure during the diastolic phase and positively correlates to the relaxing velocity of cardiac myocytes. There is a correlation between Doppler measurement of isovolumetric relaxation time and −d*p*/d*t*_max_ (*r* = 0.638, *p* < 0.001) [38]. Mainly hemodynamic determinants of −dp/dtmax are peak aortic systolic pressure and LVSP [39]. Additionally, several studies have adopted (−d*p*/d*t*_max_)/LVSP to evaluate left ventricular diastolic function [40,41]. Therefore, this study finally chose both −d*p*/d*t*_max_ and (−d*p*/d*t*_max_)/LVSP to discuss the influence of HO-1 upregulation on diastolic function more precisely. −d*p*/d*t*_max_ failed to reflect the impaired diastolic function in the CoPP untreated ISHR model, while the adjusted index, (−d*p*/d*t*_max_)/LVSP, did it. HO-1 upregulation restored both −d*p*/d*t*_max_ and (−d*p*/d*t*_max_)/LVSP, that is to say, obviously ameliorating cardiac diastolic function in the ISHR model.

+d*p*/d*t*_max_, a cardiac systolic index, stands for the maximal ascending velocity of pressure during the systolic phase and positively correlates to the contracting velocity of cardiac myocytes. There is a correlation between Doppler measurement of isovolumetric contraction time and +d*p*/d*t*_max_ (*r* = 0.842, *p* < 0.0001) [38]. Generally speaking, the ratio of +d*p*/d*t* has arrived to the maximal value before the aortic valve opens. In this way, the value of +d*p*/d*t*_max_ will not be affected by afterload. However, it will be influenced by preload and cardiac hypertrophy. According to the Frank-Starling Law, when increased quantities of blood flow into the heart, the increased blood stretches the walls of the heart chambers, leading to the more powerful cardiac contractions and the higher +d*p*/d*t*_max_ values. LVEDP indirectly stands for the preload. However, using LVEDP as the adjustment, only one study in 1993 [42] was found, and it was not convincing enough. Moreover, the value of LVEDP is usually quite low and nearly on the verge of zero when cardiac systolic function is powerful enough. So, using LVEDP as the denominator in the adjustment for +d*p*/d*t*_max_ still lacks sufficient scientific evidence. So, this study has not adopted (+d*p*/d*t*_max_)/LVEDP in discussing cardiac systolic function.

A previous study [43] has evaluated different hemodynamic indexes in ventricular contractility. The results showed that all the indexes studied were comparably sensitive to acute alterations in contractility, but no single measure can be independently used for defining an acute contractility change in the intact circulation. So, our study comprehensively analyzed different kinds of cardiac functional indexes and some adjusted values to try to get a more convincing conclusion.

To sum up, the ISHR model exhibits both systolic and diastolic dysfunction. HO-1 upregulation ameliorates the deteriorated cardiac function in the ISHR model.

#### 2.2.4. HO-1 Inhibition of Inflammatory Reaction

Inflammatory reaction is closely associated with cardiovascular diseases. Blood pressure itself and RAS activation will both activate the inflammatory process [44]. Conversely, inflammation also triggers vascular remodeling, aggravates vessel injury and exacerbates the process of hypertension and atherosclerosis [45]. So, a vicious cycle exists in these cardiovascular patients. In patients with congestive heart failure, plasma levels of pro-inflammatory cytokines were elevated, particularly TNF-α and IL-6. These increased cytokines levels, not only an epiphenomenon, but also a pathogenetic mechanism, have been demonstrated to induce myocyte apoptosis, endothelial dysfunction, left ventricular dysfunction and remodeling [6]. Although activated inflammation may provide transient protective effects in acute stage ischemia, in the long term, pathophysiological consequences of excessive inflammation are deleterious [30].

IL-6 plays various roles in driving chronic inflammation, autoimmunity, endothelial cell dysfunction and fibrogenesis [46]. In SHR, higher levels of IL-6 protein and IL-6R mRNA were observed [47]. Knockout of IL-6 was shown to attenuate AngII induced hypertension [48]. Both studies indicate that IL-6 is a hypertension pathogenic factor. IL-6 also plays a vital role in the development of pathological cardiac hypertrophy [47]. Besides, IL-6 was also reported to be associated with increased morbidity in unstable angina pectoris and depressed myocardial function in heart failure. In in-patients with acute myocardial infarction, IL-6 levels were already elevated on admittance, peaked at 1st–2nd days and remained elevated even after 12 weeks, which was highly correlated to CRP. Additionally, increased IL-6 levels after hospital discharge are associated with heart failure and depressed left ventricular function [49].

CRP, another biomarker of inflammation, is also closely related to the pathological process of hypertension and myocardial infarction. A clinical study showed that hypertensive patients had higher baseline CRP levels compared with normotensive patients. After the adjustment, an independent positive association existed between plasma renin activity and CRP levels [50], indicating that hypertension would lead to elevated CRP levels. From another angle of view, a prospective study proved that plasma CRP could predict the development of hypertension, and the future risk of hypertension was positively related to plasma CRP levels [51]. A basic experiment also manifested that overexpression of human CRP in rats caused increased blood pressure and loss of vascular endothelial nitric oxide synthase expression [52]. These results showed that, in addition to an effect on hypertension, elevated CRP was also a cause participating in the development of hypertension. Apart from hypertension, CRP has been proven to be associated with other cardiovascular diseases. For instance, in patients with AMI, serum CRP was significantly higher than in controls [53]. Moreover, CRP is also involved in the process of ventricular remodeling [32–35].

All these studies demonstrate that IL-6 and CRP are closely associated with the pathological process of hypertension and myocardial infarction, in the manner of a reciprocal causal relationship: hypertension and myocardial infarction stimulate the elevated IL-6 and CRP levels. Conversely, increased or decreased IL-6 and CRP levels also aggravate or alleviate the process of cardiovascular diseases. The inhibition of serum IL-6 and CRP levels not only reflected the attenuated inflammatory reaction, but also assisted in the suppression of infarct hypertensive pathological process. In a word, in our study, HO-1 upregulation successfully blocked the elevation of IL-6 and CRP levels, interrupted the vicious cycle and slowed the process of hypertension and myocardial infarction.

## 3. Experimental Section

### 3.1. Animal Treatment

Male spontaneous hypertensive rats (SHR) at 13 weeks (*n* = 40) and age-matched male Wistar rats (*n* = 20) were selected. After basal echocardiography and blood pressure measurement, they were divided into 6 groups: (1) WT (sham + NS (Normal Saline)), (2) WT (sham + CoPP (Co(III) Protoporphyrin IX chloride, an inducer of HO-1)), (3) SHR (MI + NS), (4) SHR (MI + CoPP), (5) SHR (MI + CoPP + SnMP (Tin Mesoporphyrin, an inhibitor of HO activity)) and (6) SHR (sham + NS), *n* = 10/group. Sham operation or coronary ligation (the ligation of the left anterior descending coronary artery ~2 mm from its emergence at the inferior border of the left atrium) were performed, respectively. The first day after operation, medications were administered among the groups: NS or CoPP 4.5 mg/kg or concurrently with SnMP 15 mg/kg, all for 6 weeks, 1/week, intraperitoneally. There was no difference in food intake in any of the treated groups.

At the 6th week, trans-thoracic echocardiography and cardiac catheterization was performed; thereafter, blood was collected for blood biochemistry, NO, IL-6, PGI_2_ testing and isolated hearts for histology and Western blot.

### 3.2. Blood Pressure Measurements

Blood pressure was measured in non-anesthetized rats with the standard noninvasive tail-cuff method.

### 3.3. Echocardiograph

*In vivo*, cardiac morphology and function were assessed by trans-thoracic echocardiography (15L8-S, H14.0 MHz) in anaesthetized rats, as previously described [54]. The LV chamber diameters were measured at the end of diastole (LVEDD) and systole (LVESD) and averaged from 3 to 5 beats. Percent fractional shortening (FS%) was calculated as follows: FS% = (LVEDD − LVESD)/LVEDD × 100.

### 3.4. Cardiac Catheterization

A catheter-tipped transducer (PE50) was introduced into the LV through the right carotid artery. Arterial and LV pressures were recorded simultaneously using a data-acquisition system (RM6240), and maximal −d*p*/d*t* (−d*p*/d*t*_max_) and +d*p*/d*t* (+d*p*/d*t*_max_) were calculated automatically from LV tracings. The LV catheter permitted measurements of LV systolic (LVSP) and end-diastolic pressures (LVEDP). After hemodynamic measurement, a blood sample was collected from the transducer into procoagulant tubes, and the serum was separated.

### 3.5. Morphology and Immunohistochemistry

Hearts were harvested and cross-sectioned. One portion was fixed in formalin, paraffin embedded and processed for histology. Tissue sections were cut to 3–4 μm thickness and processed for HE, Masson’s trichrome stain and immunohistochemistry. IOD (integral optical density) was calculated for quantitative analyses with the software “IMAGEPROPLUS”.

### 3.6. Serum TB, Glu, CRP, PGI_2_, IL-6, NO

Serum TB, Glu and CRP were tested by Hitachi-7170A Biochemical Analyzer. Serum PGI_2_ (Rat Prostacyclin, PGI_2_ ELISA kit, E90727Ra), IL-6 (Rat Interleukin 6, IL-6 ELISA kit, HE1731) and NO (Rat nitric oxide, NO ELISA kit, YH-11900E) were determined in rat serum by the enzyme-linked immunosorbant assay (ELISA).

### 3.7. Western Blot

At the time of sacrifice, other portions of heart (excluding the one for morphology) were rapidly frozen in liquid nitrogen and then stored at −80 °C. Frozen hearts were pulverized under liquid nitrogen and placed in a homogenization buffer, and protein levels were visualized by immunoblotting with antibodies against rats HO-1, HO-2 (Stressgen Biotechnologies Corporation, Victoria, BC, Canada).

Briefly, 110 μg of heart tissue lysate supernatant was separated by 12%/polyacrylamide gel electrophoresis and transferred to a nitrocellulose membrane. Immunoblotting was performed, as previously described [55]. Chemiluminescence detection was performed with the BeyoECL Plus (Beyotime Institute of Biotechnology, Jiangsu, China), according to the manufacturer’s instructions. The results were analyzed using Tanon-1600 software (Tanon1600R, Tanon Science & Technology Co., Shanghai, China).

### 3.8. Statistical Analysis

The data are presented as mean ± standard error (SEM). For comparison between treatment groups, the null hypothesis was tested by a single factor analysis of variance (ANOVA) for multiple groups or unpaired *t*-test for two groups. *p* < 0.05 was considered significant.

## 4. Conclusions

In conclusion, the ISHR model exhibited excessive inflammatory reaction, undermined endothelial function, impaired cardiac function and pathological ventricular remodeling. The pharmacological upregulation of HO-1 expression protects the ISHR model from injurious stimuli and prevents the continued deterioration in cardiac function. In consideration of the more severe clinical course in patients with both MI and HTN, HO-1 upregulation may constitute an alternative and promising approach against these plights.

## Figures and Tables

**Figure 9:**
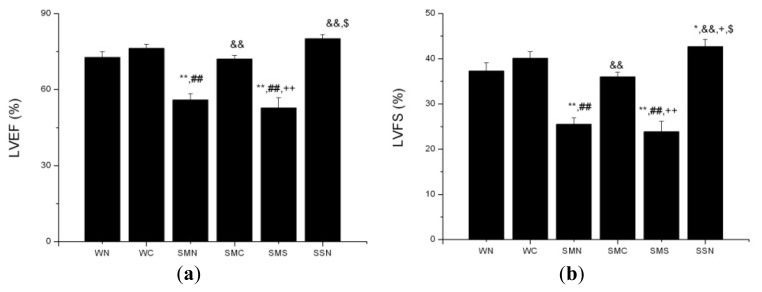
LVEF and LVFS of WTs and SHRs at sixth week after operation. (**a**) LVEF; (**b**) LVFS. *p < 0.05; **p < 0.01 *vs.* WN; ^##^*p* < 0.01 *vs.* WC; ^&&^*p* < 0.01 *vs.* SMN; ^+^*p* < 0.05; ^++^*p* < 0.01 *vs.* SMC; ^$^*p* < 0.05 *vs.* SMS. WN: WT (sham + NS); WC: WT (sham + CoPP); SMN: SHR(MI + NS); SMC: SHR(MI + CoPP); SMS: SHR (MI + CoPP + SnMP); SSN: SHR (sham + NS). *n* = 8 for each group.

**Table 1:** Baseline characters of heart rate and blood pressure.

	WTs (*n* = 20)	SHRs (*n* = 40)	*p*
HR	383.70 ± 14.174	379.69 ± 37.316	0.741
SBP	143.50 ± 198.60	198.60 ± 18.210	<0.001
MAP	124.60 ± 6.786	170.90 ± 19.503	<0.001
DBP	115.20 ± 6.374	157.24 ± 21.121	<0.001

**Table 2:** Baseline characters of echocardiographic parameters.

	WTs (*n* = 20)	SHRs (*n* = 40)	*p*
LVEDD (cm)	0.5888 ± 0.0469	0.4060 ± 0.0457	0.004
LVESD (cm)	0.3563 ± 0.03758	0.2240 ± 0.04486	0.008
LVFS (%)	40.150 ± 5.1094	44.640 ± 3.9339	0.189
LVEF (%)	76.200 ± 0.0535	81.560 ± 0.0744	0.144

**Table 3:** Histological images of WTs and SHRs in infarct and peri-infarct area. WN: WT (sham + NS); WC: WT (sham + CoPP); SMN: SHR (MI + NS); SMC: SHR (MI + CoPP; SMS: SHR (MI + CoPP + SnMP); SSN: SHR (sham + NS).

Groups	Masson (macroscopic)	Masson (×200)	Infarct area (HE ×200)	Peri-infarct area (HE ×200)
WN	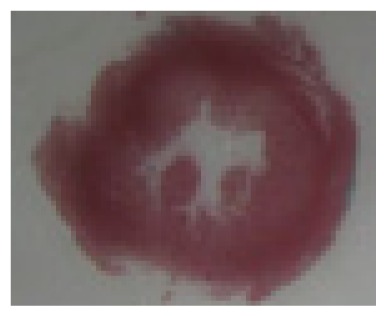	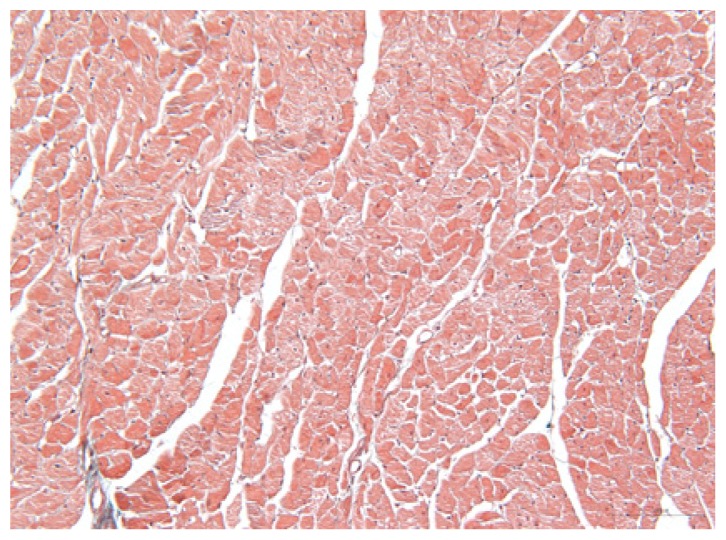		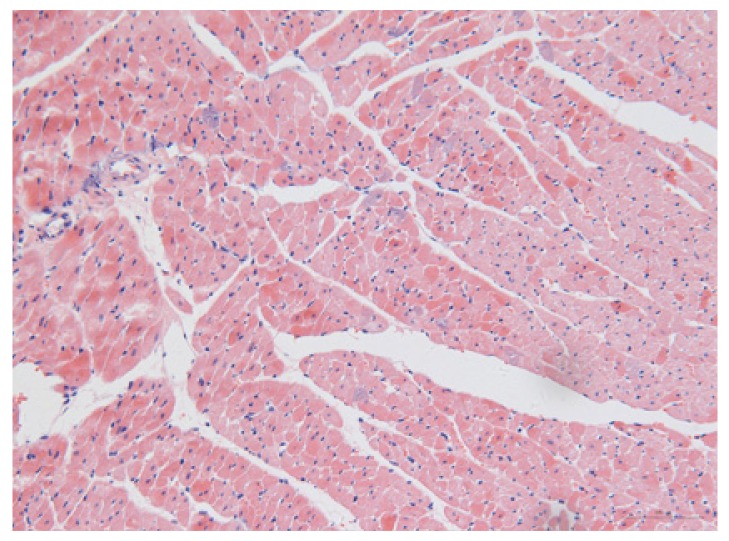
SMN	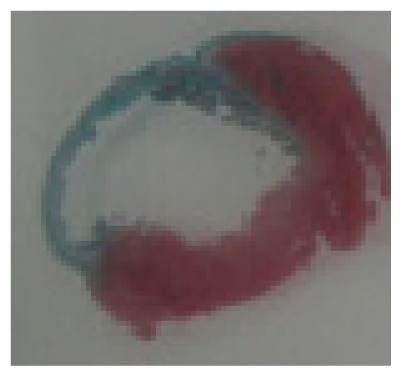	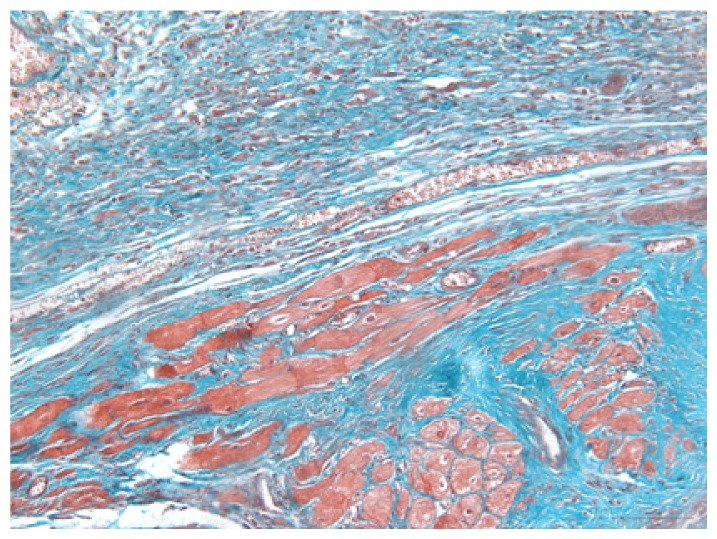	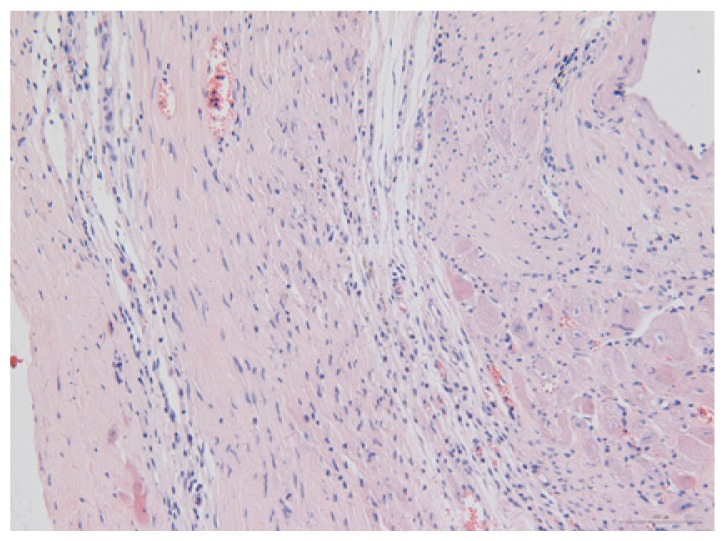	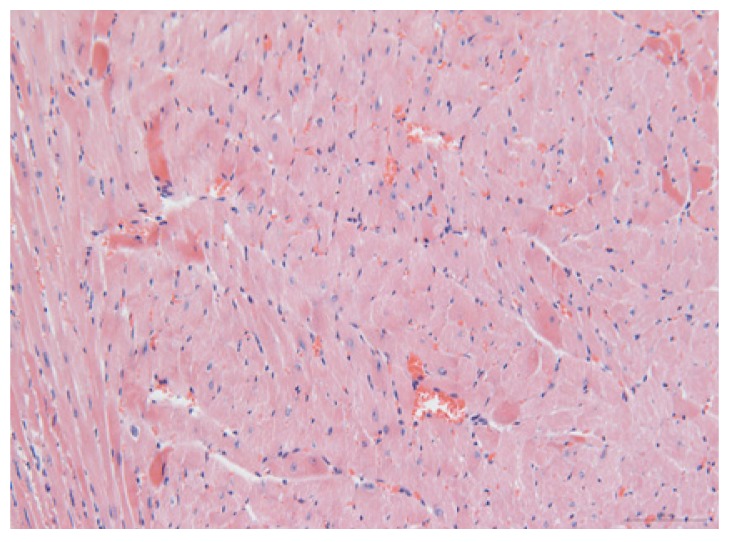
SMC	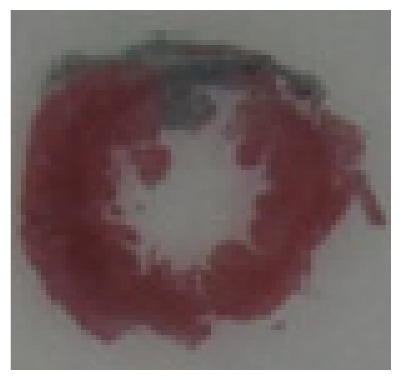	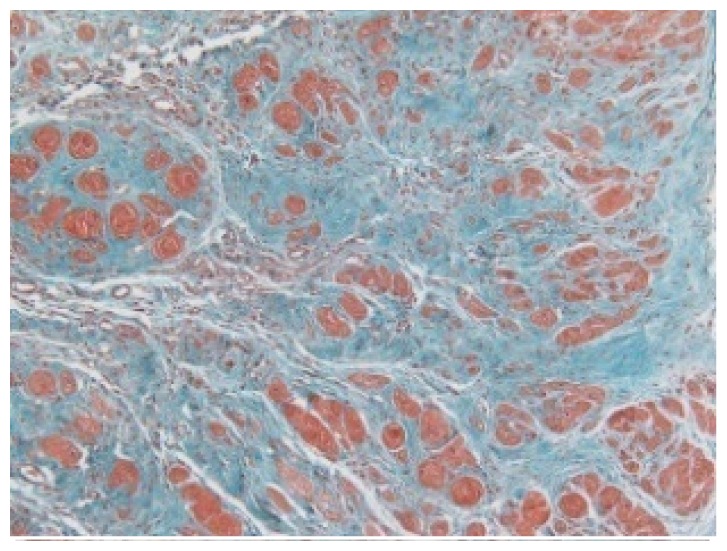	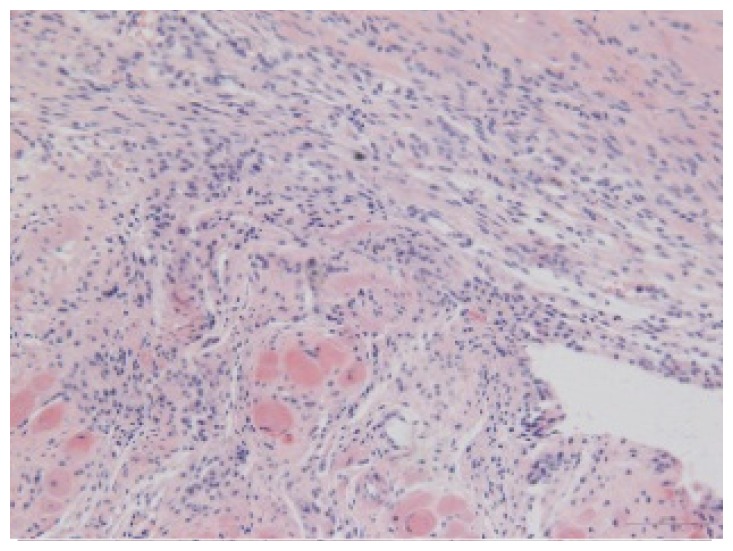	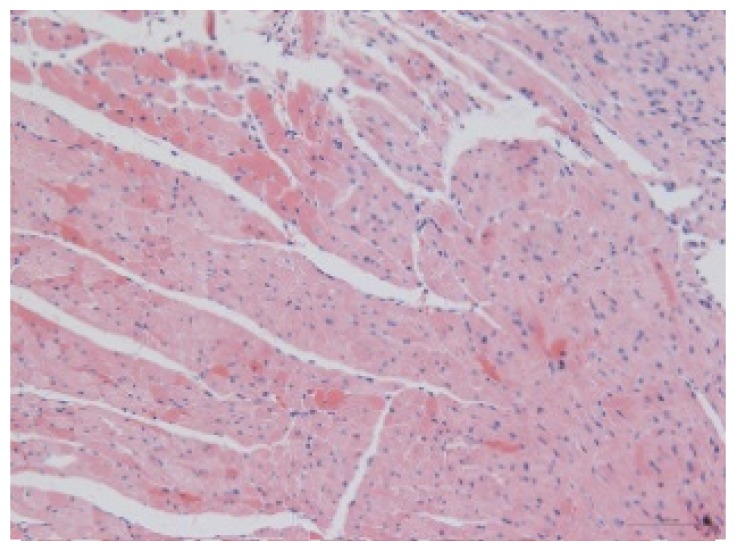
SMS	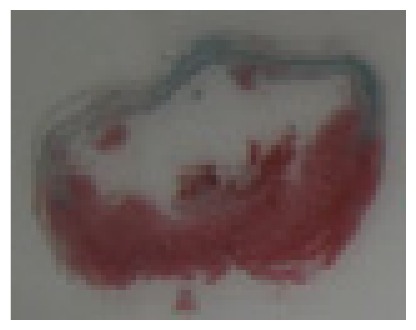	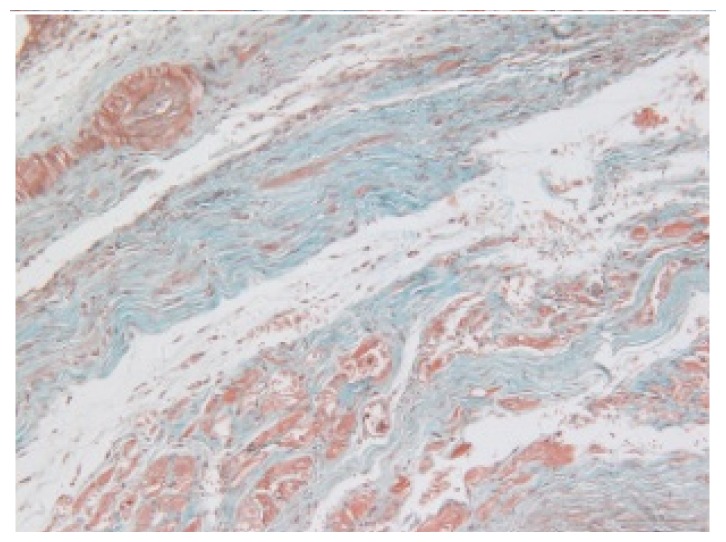	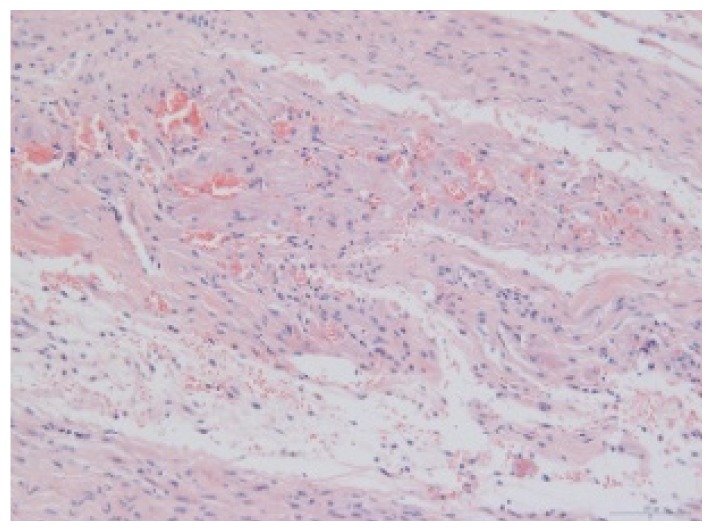	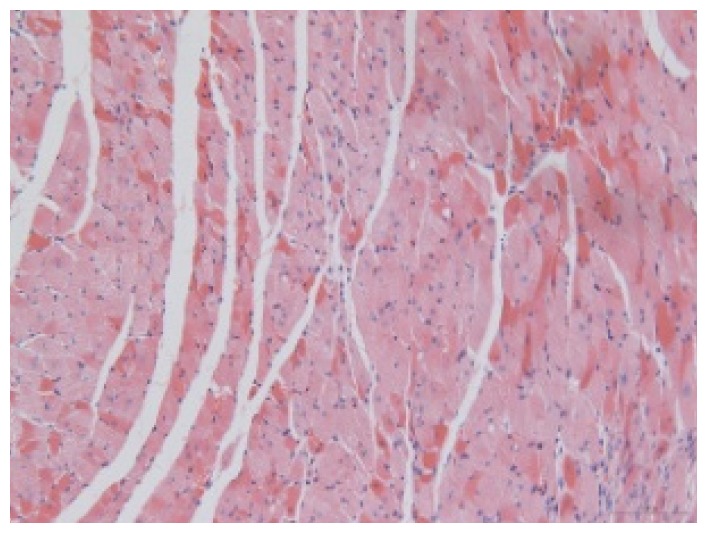

## Abbreviations

HO-1: heme oxygenase 1
CoPP: Co(III) Protoporphyrin IX Chloride
SnMP: Tin Mesoporphyrin IX Dichloride
TB: total bilirubin
WT: wistar rat
SHR: spontaneous hypertensive rat
SBP: systolic blood pressure
DBP: diastolic blood pressure
MAP: mean arterial pressure
LVEDD: left ventricular end-diastolic diameter
LVESD: left ventricular end-systolic diameter
LVSP: left ventricular systolic pressure
LVEF: left ventricular ejection fraction
LVFS: left ventricular fraction shortening
HW/BW: heart weight/body weight
TB: total bilirubin
PGI_2_: prostacyclin
NO: nitric oxide
MI: myocardial infarction
NS: normal saline
UCG: echocardiography
